# Collision Tumors of the Colon and Peritoneum: Signet-Ring Cell Carcinoma and Granular Cell Tumor

**DOI:** 10.3390/life13122263

**Published:** 2023-11-27

**Authors:** Dorela-Codruta Lazureanu, Denisa Anderco, Sorin Dema, Aura Jurescu, Remus Cornea, Octavia Vita, Bogdan Tunescu, Sorina Taban

**Affiliations:** 1 Microscopic Morphology Department-Morphopathology, ANAPATMOL Research Centre, “Victor Babeş” University of Medicine and Pharmacy, 300041 Timișoara, Romania; lazureanu.dorela@umft.ro (D.-C.L.); jurescu.aura@umft.ro (A.J.); cornea.remus@umft.ro (R.C.); vita.octavia@umft.ro (O.V.); taban.sorina@umft.ro (S.T.); 2 Pathology Department, “Pius Brinzeu” Emergency County Clinical Hospital Timisoara, 300723 Timișoara, Romania; denisa_dobre@yahoo.com; 3 Radiotherapy Department, Emergency City Clinical Hospital Timisoara, 300079 Timișoara, Romania; 4 Oncology Department, “Victor Babeş” University of Medicine and Pharmacy, 300041 Timișoara, Romania; 5 Polytrauma Department, “Pius Brinzeu” Emergency County Clinical Hospital Timisoara, 300723 Timișoara, Romania; bogdan.tunescu@gmail.com

**Keywords:** abdominal collision tumors, colon signet-ring carcinoma, peritoneal granular cell tumor

## Abstract

Collision tumors, although rare, characterized by two distinctive (morphological, as well immunohistochemical) and spatially independent tumor components at the same location, are always puzzling for clinicians, pathologists, and patients because they do not fit into the usual approaches, being neither diagnostic nor therapeutic. Reviewing the specialized literature, to date, collision tumors have been reported in multiple locations such as the skin, esophagus, stomach, intestine, liver, kidney, bladder, adrenal gland, or thyroid. We report a case of coexistence at the same site of a malignant tumor of the ascending colon and a benign tumor emerging from the peritoneal lining, initially thought by the surgeon to be right-sided serosal carcinomatosis. But histopathological examination reveals that those multiple serosal nodules were benign granular cell tumors that have collided with highly aggressive transparietal signet-ring colon carcinoma. These results put the patient’s prognosis and therapeutic strategy in a different light than the clinical and intraoperative evaluation.

## 1. Introduction

By definition, collision tumors display two distinct cell neoplastic populations developed in juxtaposition to one another without areas of intermingling. Most collision tumors occur in the crania, lung, gastro-esophageal junction, liver, rectum, bladder, uterus, and testes with two or more independent tumor components without transitional morphology [1]. Not long ago, a complex inventory of possible collision tumors was carried out, including their differentiation from composite/mixed tumors, on the organ with the largest surface of the body, the skin [2]. Recently, another case report focusing on the breast as the same site for an invasive ductal carcinoma colliding with malignant melanoma (metastatic to breast tissue) was published [3]. Within their systematic review of the literature on collision tumors of the gastrointestinal tract, Schizas et al. inventoried 53 cases over a 10-year period [4]. They evaluated the location, neoplastic types, biological behavior, and therapeutic strategy after anatomopathological diagnosis for each individual case. Most of the reported cases were in the stomach (20 cases), followed by the esophagus (17 cases), large intestine (14 cases), and small intestine, as expected, with only 2 cases.

Collision tumors are part of a wider category of mixed neoplasms together with composite tumors, carcinosarcomas, or tumors with epithelial–mesenchymal transdifferentiation, respectively, or amphicrine tumors. Although there are enough criteria for differential diagnosis between these entities, to which is added the temporal component in the treatment of the oncological patient, the terms are often used mixing the histopathological aspects with the anatomical ones and temporal consecutiveness or iatrogenic induction.

To date, within the gastro-intestinal tract, in the English literature, gastric adenocarcinomas have been described that collide with lymphomas, gastrointestinal stromal tumors, squamous cell carcinomas, and neuroendocrine tumors [5], as well as colorectal carcinomas coexisting with neuroendocrine tumors, leiomyosarcomas, lymphomas, Schwann cell hamartoma, gastrointestinal stromal tumors, or metastatic gastric adenocarcinoma [6,7,8].

The present paper highlights the coexistence in the right colon of an aggressive signet-ring carcinoma with multiple granular cell tumor nodules (Abrikossoff tumor), with the latter clinically mistaken for celomic/peritoneal dissemination (carcinomatosis). To our knowledge, this is the first time in the literature that such a neoplastic association has been reported.

## 2. Case Report

We present the case of a 60-year-old man who experiences incomplete cessation of intestinal transit for a couple of days and pain in the fossa and right abdominal flank, associated with vomiting, loss of appetite, and moderate weight loss. He addressed the Gastroenterology Clinic of the Timisoara Emergency Clinical County Hospital, where he had computerized abdominal imaging and a colonoscopy. Computer tomography highlights the thickening at the level of the ascending colon and cecum, and the colonoscopy shows a stenosing proliferative process of the right colon, with the hepatic angle not being endoscopically passable.

Local examination showed a soft, depressible abdomen that was mobile with respiratory movements, but at the level of the right iliac fossa, a hard consistency tumor mass, fixed in the deep planes, could be palpated. Because the patient’s condition worsened rapidly, he had a surgical indication, so an emergency laparotomy was decided. Intraoperatively, the cecum and ascending colon tumor was found up to the hepatic angle of the colon, with serosa retraction and posterior penetration into the retroperitoneal space. At the same time, regional adenopathies near the origin of the right and middle colic veins were identified. Right hemicolectomy with end-to-end ileotransverse anastomosis was performed.

The resected intestinal specimen—terminal ileum (10 cm), cecum, and ascending colon (together 18 cm)—was orientated in the pathological anatomy laboratory. A cauliflower tumor mass was identified from the ileocecal valve, extending for a length of 11 cm in the ascending colon, which completely stenosed the intestinal lumen and infiltrated the muscularis propria, associated with retraction of the serosa. Regardless of the ascending colon tumor, at the cecal submucosal level and in the peri-intestinal adipose atmosphere, multiple, whitish, firm, and fascicular nodules were observed and processed in the cut section.

In addition to intestinal pathology, it should be mentioned that the patient’s pathological history records a gastroduodenal ulcer with conservative treatment and lung sequelae after tuberculous infection.

After the final histopathological report, the patient received adjuvant therapy, without postoperative complications or recurrence at clinical follow-up according to oncological protocols.

The completion of the surgical procedure and the patient’s compliance to adjuvant therapy managed to prolong his survival by almost 2 years.

Tissue material (from a submitted surgical specimen submitted—orientated biopsies: tumor mass and regional lymph nodes) was processed using the standard method: 10% buffered formalin fixation, followed by paraffin embedding. The sections were stained with hematoxylin—eosin (H&E), completed with the immunohistochemical profile (IHC) (see Table 1): anti-cytokeratin 7, anti-cytokeratin 20, CDX2, S100 protein, CD117 (c-kit) and CD68, and visualization with the polymer system, using DAB (diaminobenzidine) chromogen and hematoxylin counterstain. All imunohistochemical reactions included control tissue sections, either internal or external.

### Histopathological Findings

**Figure 1:**
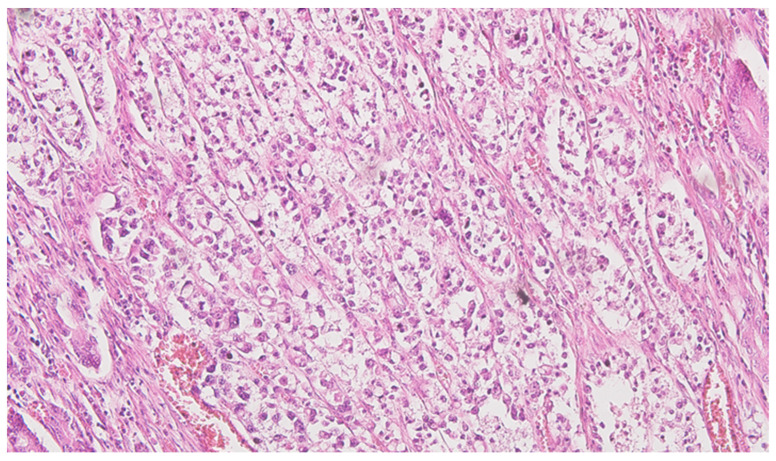
Colon mucinous carcinoma signet-ring colon carcinoma invading submucosa, HE ×200.

**Figure 2:**
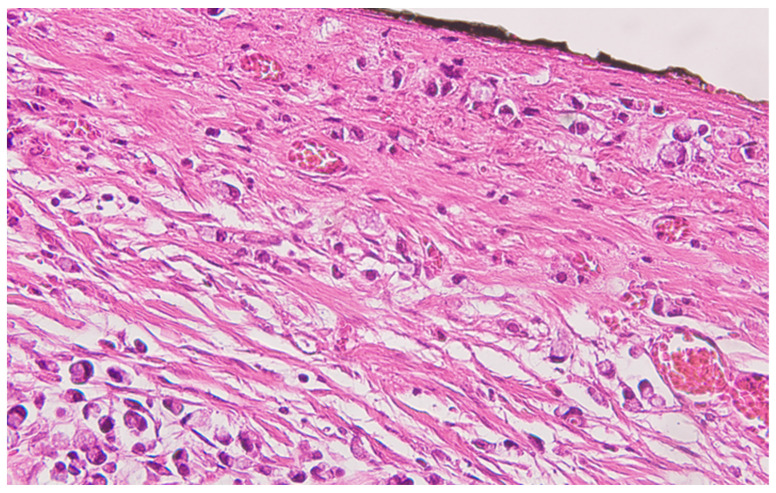
Colon mucinous carcinoma, with presence of carcinomatous cells in the subserosa, tangentially to the black ink marking of the peritoneal lining, HE ×400.

**Figure 3:**
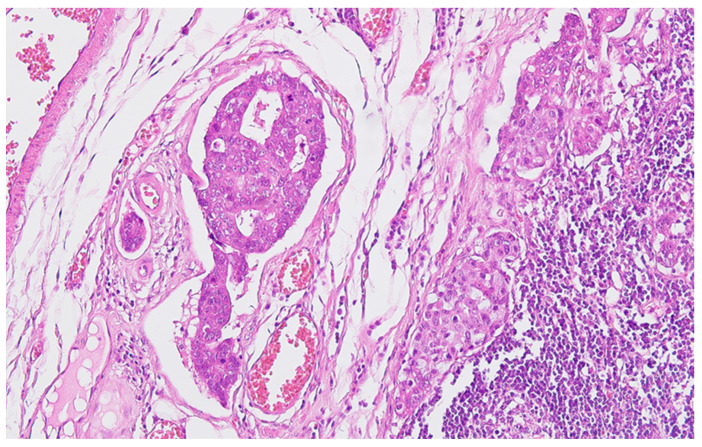
Regional lymph node metastasis of the colon carcinoma, with adenocarcinoma pattern in the afferent lymphatics and subcapsular sinuses, HE ×200.

Serial sections of the ascending colon tumor reveal massive infiltration of a diffuse, mucinous carcinoma, with predominantly intracellular secretion of mucin (“signet-ring” cells); some isolated aspects of mucinous tubular adenocarcinoma are also observed. The tumor is ulcerated on the surface, extensively invades the submucosa (Figure 1), and dissociates the muscular layer (*muscularis propria*), being found massively in the subserosa (Figure 2), with perforation of the visceral peritoneum: tumor cells in ink or less than 1 mm from the inked serosa (pT4a); numerous lymphatic tumor emboli, with frequent aspects of perineural invasion. Of 26 lymph nodes, 24 show massive carcinomatous metastases (adenocarcinoma type, Figure 3)—pN2b.

**Figure 4:**
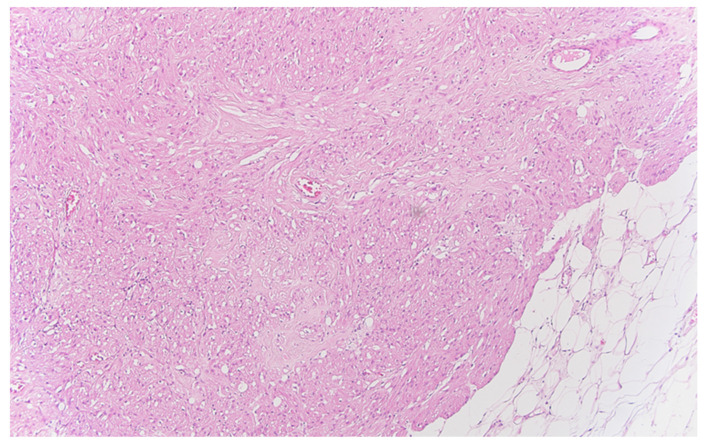
Peritoneal granular cell tumor nests and fascicles of large polygonal cells with abundant, eosinophilic, granular cytoplasm, HE ×100.

Well delineated from the previously described malignant epithelial–glandular tumor, large polygonal cell nodules, with abundant, eosinophilic, granular cytoplasm, hyperchromatic, or vesicular nucleolated nuclei are found in the submucosa of the cecum and the visceral peritoneum of the ascending colon (Figure 4); without cyto-nuclear atypia, without tumor necrosis. These tumor nodules develop in the vicinity of nerve fibers.

**Figure 5:**
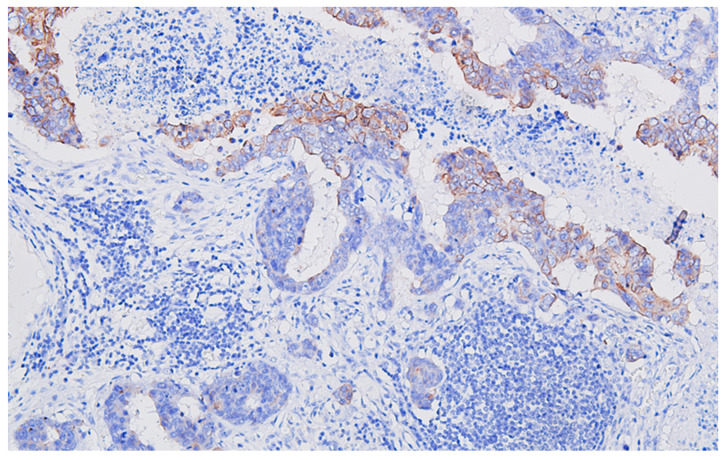
Regional lymph node metastasis of the colon carcinoma, with adenocarcinoma pattern, CK20 heterogeneous/patchy positive, DAB ×200.

**Figure 6:**
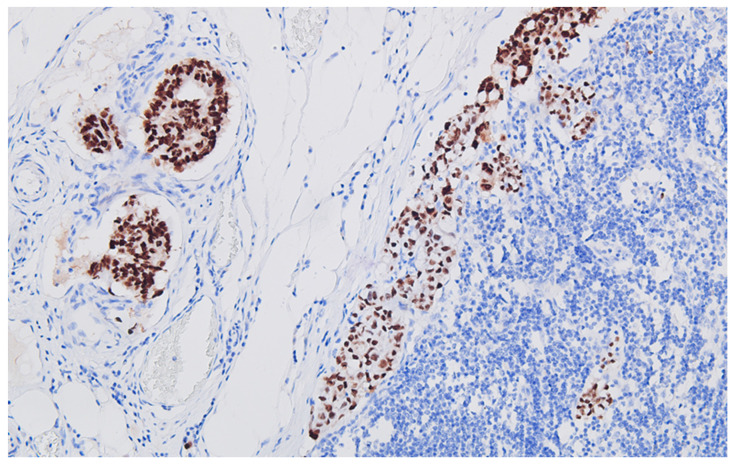
Regional lymph node metastasis of the colon carcinoma, with adenocarcinoma pattern, CDX2 positive, DAB ×200.

**Figure 7:**
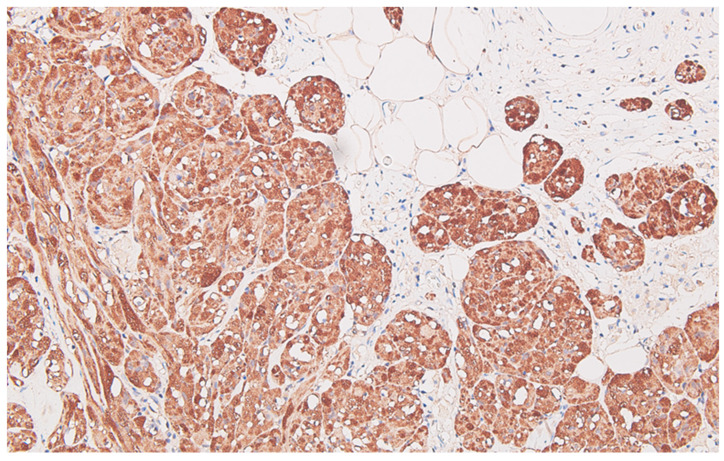
Peritoneal granular cells tumor, with intense and diffuse positive reaction for S100 protein of the neoplastic cells, DAB ×200.

**Figure 8:**
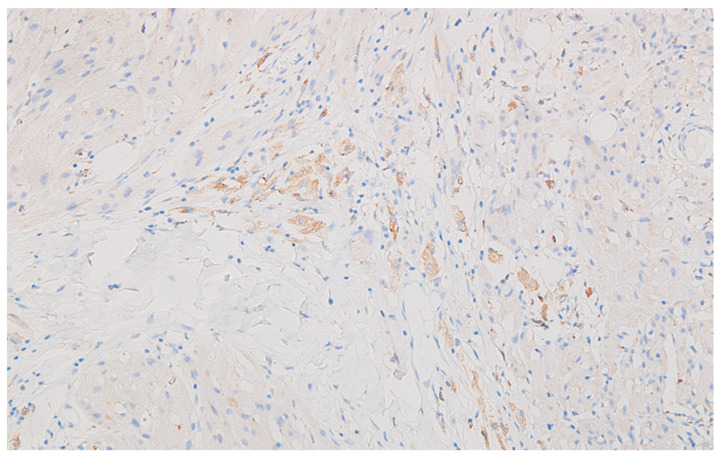
Peritoneal granular cell tumor, with faint and patchy positive reaction for CD68 of the neoplastic cells, DAB ×200.

The immunohistochemical profile of the carcinoma CK7 negative, patchy CK20 positive (Figure 5), and diffuse CDX2 positive (Figure 6) corresponds to a primary colorectal carcinoma, and for the peritoneal tumor nodules, the diffuse S100 protein positivity (Figure 7) and patchy CD68 positive (Figure 8), together with CD117 (c-kit) negative reaction, leads to the diagnosis of granular cell tumor/granular cell nerve sheath tumor/Abrikossoff tumor and excludes a possible association of a gastrointestinal stromal tumor with the colonic carcinoma.

The microsatellite stability/instability status was not evaluated in the presented case because the patient died before this expertise was validated in our health care system’s area. However, there were enough clinical and microscopic features that did not match microsatellite instability-high (MSI-high) signet-ring cell carcinomas of the colorectum: gender, absence of Crohn-like reaction, or numerous tumor-infiltrating lymphocytes (TILs).

## 3. Discussion

Because terminology can sometimes create confusion, especially when double neoplasia is called with interchangeable terms, it is better to emphasize the differences between these terms by discussing their anatomical and temporal relationships as follows:

- Collision tumors = two or more morphologically different tumors arising in the same organ, at the same time; different tumors with biological behavior, genetic, and histological features that are sharply demarcated and lack significant tissue admixture, according to Otal and Nayyar, cited by [9];
- Synchronous tumors refer to two (or more) independent primary malignancies, when the second malignancy arose within 6 months of the diagnosis of the first malignancy, in the same, or in different organs [10];
- Metachronous tumors, in which the cancers follow in sequence, that is, more than six months apart [10];
- Composite/mixed/heterologous tumors, morphological distinct tumors with cellular mixture, in the same organ; however, they have actual cellular intermingling and a common driver mutation that results in divergent histology from a common source, as proved by Aggarwal and Anani, cited by [9].

Also, the wide range of histological entities for *collision* and *composite tumors* must be highlighted: both benign or both malignant (primary or secondary—“tumor in tumor”) or one benign and the other malignant (also, primary or secondary); for *synchronous* and *metachronous tumors*, malignant proliferation is a rule [10].

Even if collision tumors are rarely reported, they always raise difficulties in diagnosis and therapeutic strategies for the patient, not to mention the huge amount of time, skill, and anatomoclinical correlation of the medical team.

Why does this event occur? There are a few explanations:

(a) From a coincidental occurrence of two primary neoplasms within a common location [9], it may be simply the chance apposition of two unrelated tumors;
(b) A double clonal origin for both components of the collision tumor [11];
(c) The two types of tumors share a common origin of pluripotent precursor stem cells that differentiate into the components of tumor cell types [12];
(d) Brandwein-Gensler et al., cited by [9], proposed another hypothesis, suggesting that the first tumor may have altered the microenvironment within the organ and increased the likelihood of developing another primary tumor or facilitated metastatic seeding within the vicinity.

In the current case, signet-ring carcinoma, by its ability to invade the perineural spaces, certainly determines the change in their microenvironment, which could trigger another clonal mutation, this time in the sheaths of nerve twigs. This change in the atmosphere of small nerves may contribute to the proliferation of Schwann cells from the myelinated sheaths of peripheral nerves at the level of the colon and its peritoneal covering.

Another particularity of the presenting case is the unexpected malignant epithelial contingent that spreads through the lymphatics: the adenocarcinomatous one, despite that in the colonic wall there was the “minor” carcinoma type as the area of involvement and the fact that it is more histologically differentiated compared to the signet-ring cell type of carcinoma. As a rule in oncology, the more differentiated the malignancy, the less eager it is to invade lympho-vascular spaces. And here it was the other way around: signet-ring cell carcinoma (the least differentiated type) was aggressive locally (visceral peritoneum perforation), and adenocarcinoma (the more differentiated type) spread to the majority of regional lymph nodes (24 positive metastases out of 26 resected lymph nodes resected). This paradoxical behavior could have led to the disturbance of the microenvironment in the invasion front of the tumor with signet-ring cells and allowed or triggered the initiation and development of the Schwann cells tumor derived from the nerve fibers in that area.

The histopathological report ruled out the clinical supposition of peritoneal carcinomatosis by unequivocally demonstrating (usual H&E stain and immune profile: S100+, CD68+) that the peritoneal nodules are composed of granular cells, without cyto-nuclear atypia, originating in the peripheral nerve sheath: granular cell nerve sheath tumor (Abrikossoff tumor). In addition, immunohistochemical profiling excluded the gastro-intestinal tumor, a very similar aspect on routine (H&E) staining with the granular cell tumor, by negative reaction to CD117 (c-kit) and positive S100, combined with patchy CD68 positive.

Between these two collided tumors, there were no intervening intermediate cell population or intermingling areas between carcinoma and granular cell tumor, the sine qua non condition for final diagnosis of the collision tumor.

The consequences of peritoneal granular cell tumor diagnosis, lacking histopathological criteria for malignancy, could be more of an academic interest or as an incidental finding after sampling due to, e.g., puzzling abdominal imaging. But its collision with colon signet-ring cell carcinoma, in an advanced stage (IIIC/pT4aN2b), definitively changed the therapeutic management and prognosis of the patient.

Even at this stage, the patient benefited from chemotherapy, especially because the surgical procedure was successful, as mentioned in several studies [13,14], but, of course, the signet-ring cell carcinoma histology *per se* signifies a dismal evolution [15,16].

The patient was followed-up with imaging and surgically for nearly 2 years after the operation without local recurrence.

The patient profile parallels that observed in the meta-analysis performed by the already mentioned Greek pathology and surgery team of researchers [4], that is, male gender and seventh decade of life. But what is completely different in the presented case is the uncommon association: signet-ring cell carcinoma with a minor adenocarcinoma component and granular cell tumor (a typical one). The meta-analysis stresses that the most common histological component was adenocarcinoma (78.6% of those 14 cases of collision tumors found published in the literature for a decade), in collision with gastro-intestinal stromal tumors or neuroendocrine tumors [4].

With regard to locally advanced epithelial malignancy, similar to the previously mentioned meta-analysis, it seems that without any connection with the size/volume of the adenocarcinoma component, precisely this component is found in regional lymph node metastases.

## 4. Conclusions

To our knowledge, this is the first time when such juxtaposition of different tumors (biological behavior and histogenesis), that of the signet-ring cell carcinoma and granular cell tumor, has been brought to the attention of medical and surgical specialists. With both being rare tumors, it was difficult to sign out the accurate histopathological diagnosis. However, all professional efforts and multidisciplinary collaboration were rewarded with the application of the appropriate therapy to the patient, increasing his disease-free survival to almost 2 years.

## Figures and Tables

**Table 1:** Characteristics of antibodies and immunohistochemical method used in our study.

Antibody	Clone	RTU/Dilution	Antigen Retrieval	Primary Antibody Incubation Time	Visualization System
CK7 Bond Leica	RN7	RTU	Epitope Retrieval Solution 1 pH 6	15′	BOND Polymer Refine Detection
CK20 Bond Leica	KS208	RTU	Epitope Retrieval Solution 2 pH 9	15′	BOND Polymer Refine Detection
CDX2 Bond Leica	EP25	RTU	Epitope Retrieval Solution 2 pH 9	15′	BOND Polymer Refine Detection
S100 Bond Leica	EP32	RTU	Epitope Retrieval Solution 1 pH 6	15′	BOND Polymer Refine Detection
CD68 DAKO	C68/684	RTU	EnVision Flex Target Retrieval Solution high pH (9)	30′	HRP Polymer Kit
CD117 Bond Leica	EP10	RTU	Epitope Retrieval Solution 2 pH 9	15′	BOND Polymer Refine Detection

RTU = ready to use; CK = cytokeratin; CDX2 = caudal type homeobox transcription factor 2; CD = cluster of differentiation.

## Data Availability

Data is contained within the article.
